# Basic science and translational implications of current knowledge on neuroendocrine tumors

**DOI:** 10.1172/JCI186702

**Published:** 2025-03-03

**Authors:** Lynnette Fernandez-Cuesta, Nicolas Alcala, Emilie Mathian, Jules Derks, Chrissie Thirlwell, Talya Dayton, Ilaria Marinoni, Aurel Perren, Thomas Walter, Matthieu Foll

**Affiliations:** 1 Computational Cancer Genomics Team, Genomic Epidemiology Branch, International Agency for Research on Cancer (IARC-WHO), Lyon, France.; 2 Department of Pulmonary Medicine, Erasmus MC Cancer institute, University Medical Center, Rotterdam, Netherlands.; 3 GROW School for Oncology and Developmental Biology, Maastricht University Medical Centre, Maastricht, Netherlands.; 4 University of Bristol Medical School, Bristol, United Kingdom.; 5 European Molecular Biology Laboratory Barcelona, Tissue Biology and Disease Modeling, Barcelona, Spain.; 6 Institute of Tissue Medicine and Pathology, University of Bern, Bern, Switzerland.; 7 Service d’Oncologie Médicale, Groupement Hospitalier Centre, Institut de Cancérologie des Hospices Civils de Lyon, Lyon, France.

## Abstract

Neuroendocrine tumors (NETs) are a diverse group of malignancies that can occur in various organs, with a notable prevalence in the lungs and gastrointestinal tract, which are the focus of this Review. Although NETs are rare in individual organs, their incidence has increased over recent decades, highlighting the urgent need for current classification systems to evolve by incorporating recent advances in the understanding of NET biology. Several omics studies have revealed molecular subtypes, which, when integrated into existing classification frameworks, may provide more clinically relevant insights for patients with NETs. This Review examines recent progress in elucidating the biology of NETs, with a particular emphasis on the tumor microenvironment and cells of origin. The existence of different cells of origin, which may contribute to distinct molecular groups, along with profiles of immune infiltration — despite being generally low — could explain the emergence of more aggressive cases and the potential for metastatic progression. Given the molecular heterogeneity of NETs and the diversity of their microenvironments and different cells of origin, there is an urgent need to develop morphomolecular classification systems. Such systems would make it possible to better characterize tumor progression, identify new therapeutic targets, and, ultimately, guide the development of personalized therapies.

## Etiology and trends in epidemiology

**Figure 1:**
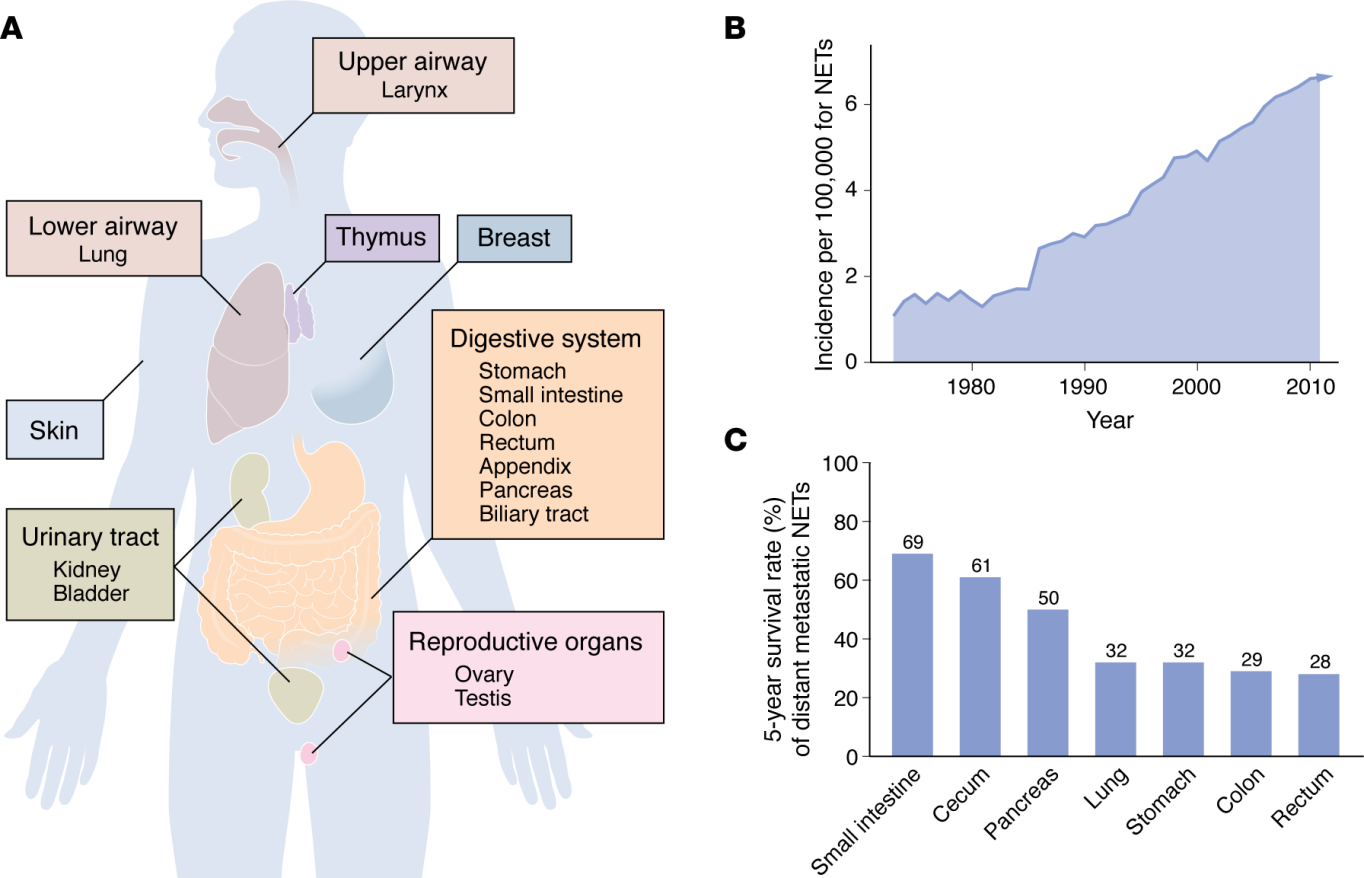
Epidemiology of NETs. (**A**) Anatomic sites where NETs originate. (**B**) Temporal trend of NET incidence in the United States from the Surveillance, Epidemiology, and End Results (SEER; https://seer.cancer.gov/) database (data from ref. 7, Supplemental Table 1). (**C**) Five-year survival rate of patients with NETs with distant metastases as a function of anatomic site (data from ref. 7, Supplemental Table 3).

Neuroendocrine tumors (NETs) are malignant, well-differentiated, epithelial neuroendocrine neoplasms (NENs) (1). NETs can be found in the upper and lower airways, thymus, digestive system, urinary tract, reproductive organs of both sexes, breast, and skin (2–6) (Figure 1A). The digestive tract and lungs are the most common sites (7–9), and this Review will focus on these. The incidence rate of NETs is around seven new cases per 100,000 people annually, and it has been rising over the past two decades (Figure 1B) (7–10). The prevalence is nearly equal between sexes, with a slight male predominance in cases affecting the digestive tract (9, 10). While most patients are in their sixties to eighties, younger individuals can also be affected, particularly in association with hereditary syndromes, appendiceal tumors, and ovarian NETs linked to dermoid cysts. NETs are rarely seen in infants and children (11, 12). The cause of NETs remains unclear. Nonetheless, hereditary genetic mutations, such as *MEN1*, *VHL*, and *NF1*, are linked to NETs in the thorax and upper digestive tract. Risk factors include a family history of cancer, older age, high body mass index, and specific risk factors shared with nonneuroendocrine cancers, such as smoking and alcohol use (13–15).

Low-grade (G1–G2) NETs usually follow an indolent course, with up to 90% of patients surviving for five years, depending on the site and stage. High-grade (G3) NETs behave more aggressively and are a recognized entity in the pancreas (16). While NETs generally have low rates of distant metastasis, those that do metastasize significantly impact the prognosis of the patients (Figure 1C). The potential to metastasize or invade nearby tissues varies based on their location, grade, and stage at diagnosis. Approximately, 20% of G1 and 50%–60% of G2 ileal and pancreatic NETs metastasize, although these numbers are a bit lower for lung NETs (9% and 25%, respectively) (17). Lung NETs have a five-year survival rate of over 90% for typical carcinoids (G1) and 60% for atypical carcinoids (G2). Pancreatic low-grade NETs have 10-year survival rates of 40%–50% (18–20). Gastroenteropancreatic NETs (GEP-NETs) have the highest rates of metastasis, with 20% showing liver metastases, followed by 5% with bone metastases and 2% with lung metastases. Lung NETs also tend to metastasize to the liver, albeit less frequently than GEP-NETs (4%–5%), and to the bones (3%). They also have a higher incidence of brain metastases compared with GEP-NETs (2% vs. 0%) (17, 21, 22).

## Current morphological classification: limitations and unfit entities

Tumor histology remains the gold standard for the diagnosis and clinical decision-making for NETs, with the WHO classification being the most powerful prognostic tool currently available (23, 24). NETs are diagnosed based on histomorphological characteristics, including organoid growth patterns and “salt-and-pepper” chromatin appearance, combined with evidence of a neuroendocrine (NE) phenotype through the expression of NE markers. However, while the NET system is used in GEP organs, the carcinoid system (typical and atypical) is still used in thoracic organs (Table 1). The grade is determined by both the proliferation rate (measured by mitotic count or Ki-67 index) and the presence of necrosis. While grading is effective for prognosis, it falls short in aiding therapy selection and identifying relapse risk. It has been shown that recurrence risk increases with higher tumor grade and TNM stage (25, 26), but establishing precise cut-offs for current markers has been challenging for GEP-NETs (27–30) and lung NETs. A recent study including over 300 samples from the lungNENomics project (31) assessed the current and emerging criteria for the classification of lung NETs using traditional pathology techniques as well as innovative deep learning approaches on whole-slide images. The authors concluded that, while mitotic criteria can be complemented by manual or automated assessment of Ki-67 or PHH3 proteins expression, these markers do not significantly improve the prognostic value of the current classification and remain highly unspecific for aggressive cases (32). In addition, there are also discrepancies in nomenclature use between different organs that impair communication among clinicians (33). For example, in the pancreas, G3-NETs are a recognized entity, while in the lung, these tumors are still termed as large-cell NE carcinomas (LCNECs) with NET-like morphology (34). It is clear that we have exhausted the potential of current morphological features in assisting the clinical management of NETs and need to investigate using further approaches. Consequently, most patients with NETs undergo surgery followed by prolonged follow-up periods (5–10 years) with conventional or functional imaging tests for monitoring (35), which are burdensome and costly. In addition, there are no defined strategies for adjuvant systemic therapies, not even for more aggressive NETs.

## Recent molecular findings and proposed molecular classifications

Although many recent molecular findings still need thorough validation, a growing body of evidence suggests that their incorporation into the current classification system via a morphomolecular classification might be more clinically relevant for patients with NETs. A summary of the molecular features of different organs is shown in Table 2.

### Lung.

Lung NETs, unlike small-cell lung cancer (SCLC) and LCNEC, exhibit a low mutation rate, with frequent alterations in chromatin-remodeling genes and rare mutations in *TP53* and *RB1* (36–38). Significant mutations include somatic inactivation of *MEN1*, *EIF1AX*, and *ARID1A*, with around 5% of patients having *MEN1* syndrome, a rare disorder that predisposes patients to developing tumors in endocrine glands and organs (39, 40). Despite being traditionally viewed as a single entity, a comprehensive multi-omic study by Alcala et al. identified three distinct molecular subgroups within lung NETs (41). Additional studies have identified similar subtypes (42, 43), or subtypes that were highly concordant with these observations (44, 45), validating the findings. Carcinoid A1 (LC1) tumors are characterized by high levels of *ASCL1* and *DLL3* expression and frequently harbor *EIF1AX* mutations. Carcinoid A2 (LC3) tumors have low levels of *SLIT1* and *ROBO1* expression. Both A1 and A2 subgroups have favorable prognoses, with more than 80% of patients surviving beyond 10 years. Carcinoid B (LC2) tumors exhibit high expression levels of UDP-glucuronosyltransferase (UGT), cytochrome P450 (CYP) family gene, *ANGPTL3*, and *ERBB4*, along with low levels of *OTP* and *TTF1*. These tumors often have *MEN1* alterations and a poorer prognosis, with only 60% of patients surviving beyond 10 years. These molecular subtypes do not align with morphological classifications but show a trend toward typical (A1 and A2) and atypical (B) carcinoids. Each subgroup has unique clinical features and potential therapeutic targets, underscoring the need for further research into their biology, risk factors, and responses to treatment. One study also reported the existence of a group of uncommon carcinoids — named supracarcinoids — that exhibit genuine carcinoid-like morphology but molecular and clinical features of highly aggressive and poorly differentiated LCNECs (41). This finding has been replicated in an independent study by Simbolo et al. (46). Further analyses are underway to better characterized this new biological entity. Emerging molecular markers, such as protein expression of CD44, ASCL1, and OTP and *TERT* gene expression have shown prognostic value in lung NETs, alongside traditional markers like Ki-67 and somatostatin receptors (47–49). Studies have proposed combining these markers to stratify patients into more clinically relevant categories than the typical/atypical classification (50). Therefore, the use of comprehensive IHC panels can further characterize these molecular groups and potentially lead to personalized treatment strategies for patients with lung NETs. Advances in nuclear medicine have also provided new methods for tumor characterization, such as somatostatin receptor targeting and metabolic imaging with ^18^F-FDG or ^18^F-DOPA (51). These approaches have led to alternative classifications and new grading schemes (52).

### Pancreas.

A large whole-genome sequencing study from Scarpa et al. provided comprehensive insights into the genetic landscape of pancreatic NETs (53). The study identified driver genetic alterations that converged into four main pathways: chromatin remodeling, DNA damage repair, activation of mTOR signaling, and telomere maintenance. The authors also found that pancreatic NETs exhibit recurrent genetic inactivation of *MEN1*, *ATRX*, and *DAXX* and the activation of the PI3K/mTOR pathway (53). Approximately 40% of sporadic nonfunctioning pancreatic NETs (tumors that do not produce hormones) harbor mutations both in *MEN1* and *DAXX* or *ATRX* (53–55). These mutations suggest that there is profound epigenetic dysregulation in pancreatic NET development. *DAXX* and *ATRX* mutations correlate with loss of nuclear expression and higher relapse risk (56). Indeed, a subgroup with recurrent loss of heterozygosity (LOH) on 10 specific chromosomes, and enriched for *MEN1*, *DAXX*, and *ATRX* mutations, showed worse prognosis and higher metastatic risk (57). Consequently, DAXX/ATRX immunohistochemistry has been suggested as a prognostic biomarker in the pancreas. In addition, the mutational status of *DAXX* and *ATRX* is strongly correlated with the alternative lengthening of telomeres (ALT), with ALT emerging as a reliable indicator of increased risk of metastasis for primary pancreatic NETs, supporting its introduction in clinical practice (58). Interestingly, once metastasized, the loss of *DAXX/ATRX* and the presence of ALT are associated with longer survival, though the reason for this is unknown (54). *TP53* and *KRAS* mutations were also shown to be more frequent in pancreatic NET metastases than in primary tumors, but *TP53* mutations were on the contrary less frequent in liver metastasis from lung NETs than in their primary counterparts (59). Insulinomas, NETs that produce insulin and are predominantly found in the pancreas, frequently exhibit mutations in the transcription factor *YY1*, which is absent in nonfunctioning pancreatic NETs (60). A notable proportion of clinically sporadic pancreatic NETs were found to have germline mutations. These include previously unreported mutations in DNA repair genes such as *MUTYH*, *CHEK2*, and *BRCA2* as well as mutations in *MEN1* and *VHL*, occurring in approximately 17% of patients (53).

For G3 pancreatic NETs, the limited data indicate that these tumors often harbor mutations in *DAXX/ATRX* and *TP53*, along with the loss of *RB1* (61). Their epigenetic profile appears to be similar to that of G1 and G2 tumors (62). Notably, progression from G2 to G3 is not uncommon. While this progression can occasionally occur in naive tumors, it is more frequently observed under therapy (63–65).

Several transcriptomic analyses have proposed various molecular subclassifications, yet their clinical implications remain unclear. Initial analyses identified three main subtypes of pancreatic NETs, including less aggressive insulinomas and two nonfunctional clusters associated with metastatic disease (66). The metastasis-like primary 1 (MLP-1) and MLP-2 subtypes share gene signatures related to fibroblasts, stem cells, and hypoxia, with MLP-1 showing an immune-suppressive profile (67). These profiles could pave the way for new immunotherapy approaches for these tumors. Scarpa et al. also identified a subgroup of tumors associated with hypoxia and HIF signaling, suggesting that stemness, hypoxia, and metabolic changes, along with immune profile alterations, are major phenotypes associated with aggressive pancreatic NETs (53). However, transcriptome profiles do not clearly distinguish between small indolent tumors and those with a high relapse risk. Despite the low mutational burden (68), the frequent mutations in chromatin regulation genes highlight the significant role of epigenetic dysregulation in pancreatic NETs. Chromatin immunoprecipitation assays identified two major subtypes of pancreatic NETs based on H3K27ac enhancer profiles, one with an α signature expressing *ARX* and one with a β signature expressing *PDX1* (69). DNA methylation profiles further stratify tumors by cell of origin, genetic background, and prognosis, identifying α-like tumors (*MEN1* mutated and indolent) and intermediate tumors (*MEN1* and either *DAXX* or *ATRX* mutated and with high risk of relapse), suggesting a progression from α to intermediate tumors (70).

### MEN1 in pancreatic and lung NETs.

A common occurrence among pancreatic NETs and lung NETs is the presence of both sporadic and germline *MEN1* mutations. MEN1 is associated with histone lysine methyltransferase activity and regulation of the cell-cycle pathway through CDKN1B (p27) and CDKN2C (p18). In lung NETs, the identification of a somatic *MEN1* mutation correlates with a poorer prognosis (41, 71), though it may be indolent in α-like and sporadic pancreatic NETs with isolated MEN1 deficiency (65). When combined with a *DAXX* or *ATRX* mutation, *MEN1*-inactivated pancreatic NETs have a poor prognosis. Interestingly, patients with germline *MEN1* mutations and lung NETs have a favorable prognosis, possibly owing to early screening that identifies 86% of tumors, allowing timely curative treatment (72, 73). The heritability of *MEN1* alterations among siblings of patients with MEN1-related lung NET and pancreatic NET is lower compared with other NET types (pituitary, adrenal, thymic) (74), though the reason for this is unclear. Thus, while MEN1 is clearly linked to the development of pancreatic and lung NETs, its precise role in tumor aggressiveness across different anatomical sites and its molecular mechanisms require further investigation.

### Small intestine.

Small-intestinal NETs also have a very low mutational rate, with the only recurrent mutations occurring in the *CDKN1B* gene in 8% of cases and loss of chromosome 18 observed in 50%–80% cases (75, 76). Three molecular subtypes have been described based on DNA methylation and genetic alterations. One group harbors chromosome 18 LOH (18LOH group, 55% of tumors), another group shows no large copy number variations (19%), and the third group exhibits multiple copy number variations, including gains of chromosomes 4, 5, and 20 (26%). Notably, tumors with *CDKN1B* mutations are found within the 18LOH group. A difference in progression-free survival was identified among these three subgroups, with the 18LOH group having longer progression-free survival compared with the other two groups (77). Additionally, small intestine NETs also exhibit DNA methylation clusters associated with different prognoses, CNV variations, and genetic backgrounds (77).

## Cell of origin and microenvironment in NET development

### Cells of origin.

The heterogeneity of NE cells throughout the body, including pancreatic islet cells and enteroendocrine cells in the intestine, is well established. Each of these NE cell populations is subdivided into specific subtypes primarily defined by the hormones they express. There is emerging evidence of heterogeneity among NE cells in the lung (78–81). Beyond hormonal or neuropeptide expression, heterogeneity may also arise concerning innervation (82). These cells might have different functions compared with the noninnervated ones. Furthermore, a third level of heterogeneity in NE cells can be attributed to their spatial distribution within tissues. For example, NE cells in the trachea likely differ in function from those in the bronchi or bronchioles in the lung, with the latter usually consisting of multiple NE cells clustering as a NE body. In the lung, tumorlets (small carcinoid lesions <5 mm) are considered preneoplastic lesions. These tumorlets are abundant in a condition called diffuse idiopathic NE cell hyperplasia, which is generally diagnosed in older female individuals with peripheral nodules and seems to be associated with the A1 molecular carcinoid subtype and *EGFR* expression (49, 83). NE cell hyperplasia may also develop more centrally in the airways and could contribute to the formation of centrally located carcinoids, which seems to be more often observed in younger patients and linked to the A2 molecular subtype. Therefore, the spatially distinct locations of NE cells may correlate with different molecularly defined lung NET subtypes, warranting further investigation.

Most NETs originate from NE cells of epithelial origin. These cells arise from local pluripotent stem cells in the endoderm, which undergo NE differentiation under the influence of transcription factors like *PDX1* and *NGN3* in the GEP system and *ASCL1* in the lung. Examples include NETs from enterochromaffin cells in the gut and insulinomas from pancreatic islet cells. Insulinomas are hypothesized to originate from β cells in the islets of Langerhans, as suggested by epigenetic data (84). Genetic mutations in nonfunctioning NETs differ from those in insulinomas, indicating varying susceptibility among different cell types (85).

Owing to the scarcity of NE cells in most organs, our understanding of their subtypes and their association with specific tumor types remains limited. For example, a recent single-cell cancer atlas of the lung identified just 500 NE cells of 2.4 million lung cells, accounting for only 0.02% of the total (86). Several lines of evidence highlight the pivotal role of the cell of origin in determining tumor type following specific oncogenic mutations, particularly for SCLC (87, 88) and GEP-NEC (89, 90). But cells of origin have not been as extensively explored in NETs, partly due to the limited number of available models.

Most NETs exhibit low levels of immune infiltration but may present heterogeneous immune subtypes (41, 91), alongside significant abnormal vascularization (92, 93). Supporting these observations, a recent small single-cell study of three lung NETs revealed a distinct microenvironment characterized by the presence of noninflammatory monocyte-derived myeloid cells, vascular smooth muscle cells, pericytes, and a small proportion of potentially prognostic cancer-associated myofibroblasts (94). Transcriptomic analyses have also found lung supracarcinoids to have high infiltration levels (41). These tumors exhibited increased expression of immune checkpoint genes such as *PDL1*, a phenomenon observed occasionally in a small subset of NETs across various sites (95, 96). The microenvironment of pancreatic NETs shows a lower density of immune cells (97) than that observed in pancreatic NECs. An increased presence of CD8^+^ T cells combined with a reduced number of macrophages is associated with better outcomes (98, 99).

**Figure 2:**
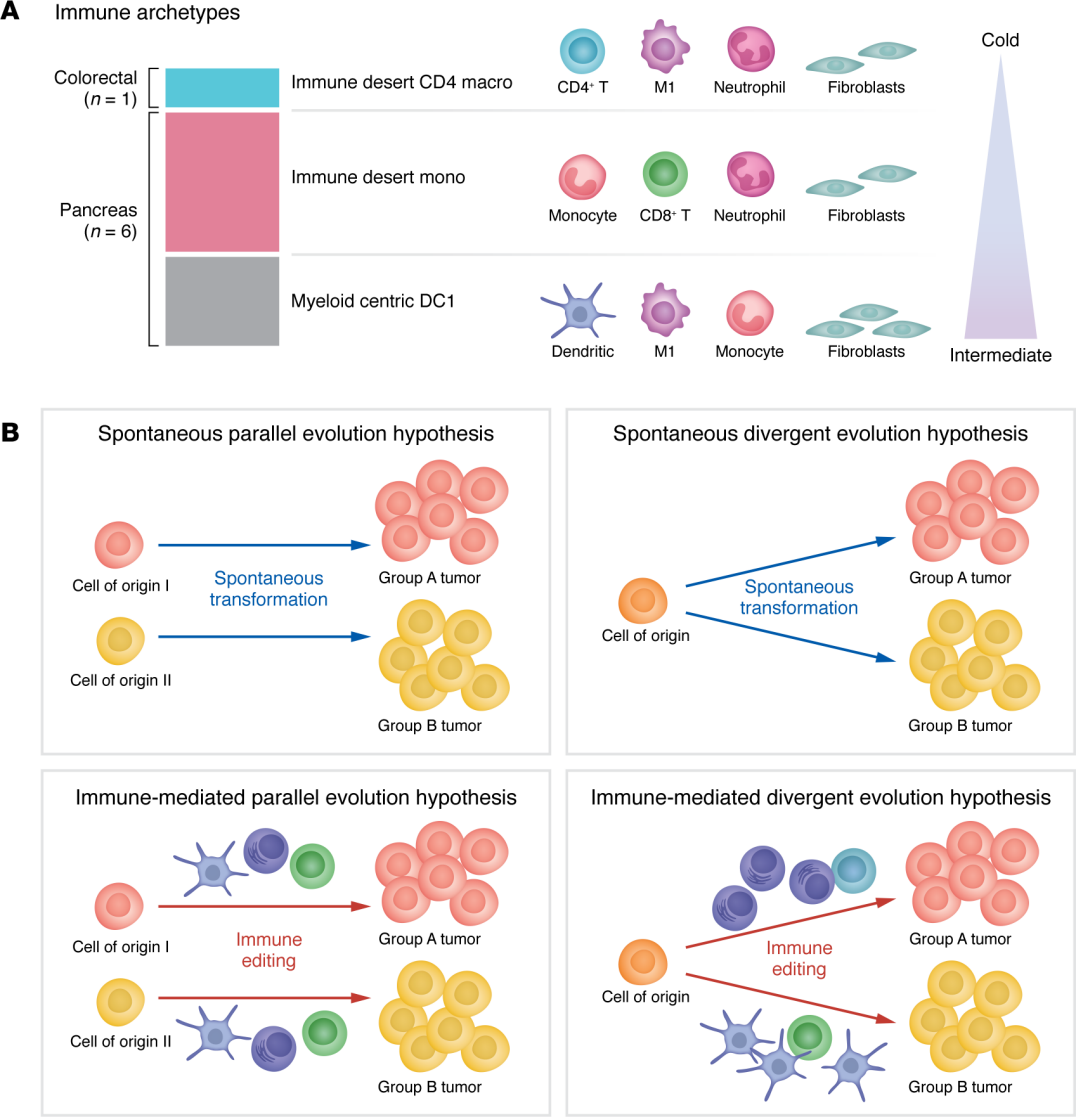
The microenvironment of NETs. (**A**) Immune archetypes observed in NETs. Data shown are from Combes and colleagues (101). The hot-intermediate-cold classification of archetypes corresponds to that of Galon et al. (163), as reported Combes and colleagues (100) (representation of archetypes adapted with permission of Springer Nature Limited, which retains rights to the reference image). (**B**) Competing hypotheses about the crosstalk between the cell of origin of NETs and their microenvironment. (Top left) Under the spontaneous parallel evolution hypothesis, the cell of origin is solely responsible for the observed tumor groups (histopathological or molecular types and subtypes), and crosstalk between NE cells of origin and their microenvironment does not influence carcinogenesis. (Top right) Under the spontaneous *divergent* evolution hypothesis, somatic alterations (genetic or epigenetic) are responsible for the observed tumor groups, putting the NE cells of origin on different evolutionary trajectories. (Bottom left) Under the immune-mediated parallel evolution hypothesis, the cell of origin determines the observed tumor groups, but crosstalk between NE cells of origin and their microenvironment is crucial to initiate carcinogenesis. (Bottom right) Under the immune-mediated *divergent* evolution hypothesis, different microenvironments are responsible for the observed tumor groups by induction of different selective pressures that put the NE cells of origin on different evolutionary trajectories.

Recent pan-cancer analyses have classified immune microenvironments into 6–12 dominant archetypes, which may share evolutionary traits and vulnerabilities, particularly in relation to immunotherapy (100, 101). Among these archetypes, several immune-desert and myeloid-centric environments might be pertinent to NETs, although the very small sample size for NETs (*n* = 7) allows only for the formulation of hypotheses that require further validation in large cohorts, and the link with known NET molecular subtypes has not been explored. Specifically, pancreatic NETs (4 G1, 1 G2) and a colorectal (G1) NET were categorized as either immune-desert or myeloid-centric archetypes (101) (Figure 2A). The immune-desert monocyte archetype found in pancreatic NETs is associated with higher rates of cancer-associated fibroblasts, aligning with preliminary single-cell data on lung NETs; the only colorectal NET included in the study presented another immune-desert archetype, biased for CD4^+^ T cells and macrophages. Myeloid-centric environments can be mistaken for immune deserts due to the lack of T cells, explaining why NETs are often considered uniformly “cold” tumors. The myeloid-centric type 1 dendritic cell (DC1) archetype is characterized by elevated levels of type 1 conventional dendritic cells, which might also occur in subsets of lung NETs, in particular in the A1 molecular group, based on indirect evidence from transcriptomic deconvolution (41). This archetype may also feature higher levels of neutrophils compared with other immune archetypes. The existence of lung NETs with high infiltration, in particular in supracarcinoids (41), suggests that some tumors may correspond to the immune-rich or immune-rich stromal archetypes, although the precise archetype remains unclear.

### Interaction between cell of origin and microenvironment.

NE cells from different tissues show evidence of dynamic crosstalk with their microenvironment, such as the response of NE cells to environmental and cellular derived stimuli and the direct influence they can exert both locally and systemically through the bioactive compounds they secrete (91). However, the interactions between the tumor microenvironment, NE cells, and tumor cells during carcinogenesis and NET progression are not well understood, resulting in unproven hypotheses (Figure 2B). The limited level of immune infiltration is generally interpreted as evidence of the limited impact of immune infiltration on NET evolution. Nevertheless, there is evidence that both immune-desert and myeloid-rich archetypes observed in pancreatic NETs and a colorectal NET strongly influence tumor formation and progression. The myeloid-rich DC1 archetype is speculated to evolve from a fibroblast-macrophage-monocyte axis. It exhibits features similar to those of visceral adipose tissue, where Tregs interact with macrophages to regulate adiposity. In this environment, conventional dendritic cells acquire a tolerogenic phenotype with decreased antigen-presenting functions. This phenotype is sustained by PPARγ, IL-10, and steroid signaling (100). The immune-desert monocyte archetype is speculated to resemble the immune system’s response during midstage wound healing. This stage is characterized by the presence of few T cells but many neutrophils and immature myeloid cells. This pattern aligns with skin wound healing, where neutrophil and monocyte infiltration is initially driven by IL-1 and TGF-β. Subsequently, monocytes differentiate into macrophages, which clear cellular debris and interact with fibroblasts to promote tissue healing through extracellular matrix remodeling (100). Interestingly, immune-desert and myeloid-centric tumors had the highest expression of Ki-67 and of cell-cycle–associated genes in general, suggesting that their lack of T cells might be associated with cell-cycle checkpoint avoidance.

## Cell plasticity and disease progression

Little is known about the progression from primary to metastatic in lung and pancreatic NETs. Most studies focus on changes in proliferation rates, with over 35% of lung NET and 50% of pancreatic NET metastases showing increased rates compared with primary tumors (22, 102). Few studies assess molecular changes in consecutive specimens, but low genomic heterogeneity between primary tumors and synchronous liver metastases has been observed (103). However, other studies report increased genomic imbalances in metastases (104). Mechanisms associated with metastasis formation include hypoxia, metabolic changes, and a stem-cell-like phenotype (66, 67). A recent study identified immune escape, stem cell signaling, and cell reprogramming as key pathways in progression (105). Additionally, an increased number of tumor-infiltrating T cells in metastatic pancreatic NETs suggests immune pathway activation (106). Comparing primary and metastatic transcriptomes has revealed potential treatment targets, though underlying progression mechanisms remain unclear (107). In the case of small intestine NETs, DNA methylation appears to change progressively from normal tissue to primary tumor and then to metastasis, suggesting that tumor evolution may be driven by epigenetic mechanisms (108).

In addition to well-differentiated NETs, NENs also include poorly differentiated and more aggressive NECs. NE cells are believed to be the origin of most NENs. Their plastic and dynamic nature is highlighted by their ability to respond to external stimuli and microenvironmental signals in a context-specific manner. NE cells of the stomach, intestine, pancreas, and lung have all been shown to be altered in number under certain pathological conditions. However, although they share characteristics, NETs and NECs are usually considered separate entities with distinct evolutionary histories and cells of origin (84, 109, 110). Indeed, normal epithelial cells can directly acquire a small-cell NEC phenotype by acquiring drivers that can reprogram cells (111). Balanis et al. also identified a convergence to a small-cell NE state across various epithelial cancers, often linked to poor prognosis (112). Similarly, treatment is known to influence the transition toward NEC (113), for example, adenocarcinoma of the lung and prostate under prolonged exposure to EGFR blockade or treatment-induced suppression of the androgen receptor in prostate cancer (114–117). The specific genomic profile of NECs across organs, almost always *TP53* and often *RB1* mutations, especially in SCLC (37, 84), also suggests fundamental differences between NETs and NECs and the necessary mechanisms for a convergent NEC phenotype. Nevertheless, although concurrent inactivation of *TP53* and *RB1* in prostate cancer was shown to lead to a NE phenotype in preclinical models (118), similar inactivation did not lead to the expression of NE markers in the colon (89), and it seems the situation might be the same in the lung. The existence of *TP53* and *RB1* in lung adenocarcinoma further supports the fact that these two genes might not be sufficient for NEC formation in all organs (119). It should also be noted that there is speculation of transdifferentiation from NETs to adenocarcinoma in the pancreas, in particular related to the acquisition of *KRAS* mutations (59).

However, there is some evidence suggesting that the separation between NETs and NECs might be subtler and that the progression of NETs toward a more aggressive NEC-like molecular profile exists. The discovery of supracarcinoids (41), the existence of which was confirmed in other cohorts (43, 46), further supports this link. Although rare, these highly aggressive entities resemble NECs, making it crucial to understand them for identifying patients with poor prognosis and specific treatment needs as well as to explore the link between NETs and NECs. The absence of typical NEC alterations in these samples suggests phenotypic convergence toward NECs through alternative drivers. Their unique microenvironment, composed of myeloid cells and macrophages, like that of LCNEC rather than NETs, may contribute to the ecological niche necessary for this convergence (120). One mechanism through which lung NETs may acquire NEC features is chromothripsis, typically affecting chromosomes 3, 11, and 12 (41, 53, 121). This phenomenon has been identified in up to 3% of SCLCs, primarily in never-smokers, and is associated with a unique molecular profile characterized by intact *RB1* and *TP53* genes. These tumors were enriched for mutations in cell-cycle gene *ATM* and chromatin remodeling genes *ARID1A*, *MEN1*, and *EIF1AX*, which are most observed in lung NETs (36). Some patients with this profile were indeed later identified as having lung NETs based on a different tumor sample. The increased oncogenicity was partially attributed to cell-cycle pathway disruption through amplification of *CCND1* or *CCND2/CKD4/MDM2*. Notably, similar amplification of chromosomes 3 and 11, along with enrichment for *ATM* mutations, has been previously observed in lung NETs/LCNECs with higher proliferation rates (122).

Similar evidence is observed in pancreatic NETs. During progression, α-like tumors can evolve into intermediate tumors upon mutations in *DAXX* and *ATRX*, leading to genomic instability and activation of ALT (70). Rarely, metastatic insulinomas show *ARX* positivity with concurrent loss of *DAXX/ATRX* and ALT activation, suggesting a distinct tumorigenic mechanism in malignant insulinomas similar to nonfunctional pancreatic NETs through transdifferentiation from α cell tumors (123). In a *Tp53-* and *Rb*-mutated mouse model, metastatic and primary tumors arise from low-grade insulinomas via dedifferentiation along the β cell developmental pathway, resulting in downregulation of mature β cell markers and expression of pancreatic progenitor markers (124).

## Biologically driven therapeutic opportunities

Among the systemic therapies for metastatic NETs, two somatostatin analogues (SSAs) (125, 126) and one peptide receptor radionuclide therapy (^177^Lu-edotreotide) (127) are accepted (Table 3). Regarding molecularly targeted therapies, everolimus (a mammalian target of rapamycin mTOR inhibitor, refs. 128–130), three tyrosine kinase inhibitors with antiangiogenic activity (sunitinib for pancreatic NETs, refs. 131; surufatinib in China, refs. 132, 133 for all NETs; and probably soon cabozantinib, ref. 134), and belzutifan (for patients with von Hippel–Lindau (VHL) disease, targeting HIF-2α, ref. 135) have been approved. Systemic cytotoxic chemotherapies include alkylating agents such as streptozotocin, temozolomide (combined with capecitabine), dacarbazine, and oxaliplatin.

Despite the several prognostic factors available for NETs management, almost no predictive factors of response to a specific treatment are prospectively validated (136, 137). Except for ^177^Lu-edotreotide, which requires the expression of somatostatin receptor 2 (SSTR2) on somatostatin receptor imaging, and belzutifan, which requires a germline mutation in the *VHL* gene, all other treatments are prescribed without driver biomarkers. Promoter methylation or low expression of methylguanine-methyltransferase (*MGMT*) is the best-proven predictive factor of response to alkylating agents including temozolomide; evaluating its status could help in choosing chemotherapy for NETs (138, 139). However, the assessment of *MGMT* methylation and expression by validated platforms must become more widely available before larger clinically use.

Through recent advances in our understanding of the molecular alterations occurring in NETs, the options for molecularly targeted therapy of driver mutations remain limited. This is because some NET subtypes harbor very few molecular alterations (small intestine NETs) and the more frequently occurring alterations are not actionable (*MEN1, DAXX/ATRX,* and *ARID1A* in pancreatic and lung NETs). Subsequently, achieving effective personalized medicine in NETs remains a challenge compared with other cancers. In a recent study from Boilève and colleagues, 19 patients (four with a NEC, 15 with a NET) were treated with molecularly targeted therapy as follows: immunotherapy (*n* = 3), tipifarnib (*n* = 1), NOTCH inhibitor (*n* = 1), EGFR inhibitor (*n* = 2), HER2 inhibitor (*n* = 1), and everolimus (*n* = 11); clinical benefit was seen in 67% of cases (140). In addition, positive outcomes have been seen in rare subtypes of NETs treated with an agnostic approach targeting rare gene rearrangements, including *ALK*, *ROS*, *RET*, *NRG1*, and *NTRK1* (141, 142). The prevalence of these molecular alterations may be associated with the organ of origin. For example, *BRAF-V600E* mutation is more common in colon NECs than in colon adenocarcinoma (143), and it has also been identified in a supracarcinoid (143). In lung NETs, the presence of a targetable driver (e.g., ALK or RET) may be associated with a mucin-enriched or combined adenocarcinoma tumor, warranting further investigation (145, 146). The discovery of gene fusions and oncogenic driver mutations in a subset of NETs underscores the importance of genomic evaluation in these tumors. Finally, using “liquid biopsy” to identify the predominant NET clones to determine actionable alterations may help identify patients for personalized medicine (147, 148).

In lung NET, new insights may indicate drug susceptibility according to epigenetic/transcriptional molecular subtypes. The recent observation that some lung NETs express EGFR and that lung NET patient-derived tumor organoids require EGF for their growth suggests the need for clinical studies to determine whether EGFR could be a predictive biomarker for the response of a subset of lung NETs to EGFR-targeted therapies (144). In addition to EGFR, the expression of delta-like ligand 3 (DLL3), an inhibitory Notch pathway ligand, has been identified in lung NETs (149). DLL3 is significantly higher expressed in A1 but not in A2/B lung NET subtypes (41, 49). Proof of concept was recently highlighted in a case report of a patient with atypical lung NET showing clinical efficacy on treatment with a DLL3 bispecific T cell engager (150). Finally, a subset of lung NETs also shows high expression of hepatocyte nuclear factor 1α and 4α (HNF1a/HNF4a). In vitro such HNF^+^ lung NETs have been correlated with a response to FGFR3 and FGFR4 inhibitors (45).

There is evidence suggesting that most of these approved drugs also target the tumor microenvironment in addition to NET cells. SSAs, a cornerstone in NET management, bind to SSTRs overexpressed on many NET cells, inhibiting the release of various hormones and growth factors that alter the microenvironment. Therefore, SSAs are effective not only in controlling secretory syndrome induced by NETs, but also in stabilizing the disease with an antiproliferative effect (151). NETs often exhibit high levels of *VEGF*, which promotes angiogenesis by binding to *VEGFR* on endothelial cells. Key signaling pathways, such as PI3K/AKT/mTOR and Notch, regulate angiogenesis in NETs. Therefore, VEGF inhibitors, like sunitinib (131), cabozantinib (134), and bevacizumab, and mTOR inhibitors, like everolimus, which target angiogenesis, are potential treatments (130, 152). Understanding the molecular mechanisms of angiogenesis in NETs is essential for developing effective new drugs. Belzutifan is a novel agent targeting the HIF pathway, which plays a crucial role in cellular response to hypoxia and is dysregulated in some NETs (135). Though its current indication is in patients with VHL disease, belzutifan’s mechanism should also be identified outside the rare situation of germinal *VHL* disease.

Regarding other treatments targeting the microenvironment, immunotherapy using checkpoint inhibitors (CPIs) alone has been disappointing in NETs; this is especially due to its lack of predictive factors of response (153). Nevertheless, there is a subpopulation of NETs that show a signal of susceptible for response to CPI, enriched for atypical lung carcinoids (153–156). Tumor mutational burden and microsatellite instability–high status have been associated with better responses to CPI, as they may increase neoantigen load, enhancing immune recognition. However, they remain rare (<5%) in NETs (157). PD-L1 expression seems less predictive for response to CPI in NETs than in other cancers (153). Whether temozolomide-induced high tumor-mutation burden is a suitable molecular selection marker for immunotherapy in NETs is yet to be determined, as conflicting data have emerged from glioblastoma and colon cancers (158, 159). Combining immunotherapy with targeted therapies or peptide receptor radionuclide therapy may enhance its effectiveness and overcome resistance mechanisms, but the first results reporting this association were conflicting (160, 161).

## Unanswered questions and future directions

The nomenclature gap across organs seems to be slowly closing, as in 2022, the WHO proposed a uniform NET nomenclature (1, 162). Still there is an urgent need for a clear, clinically relevant definition of high-grade NETs that incorporates current concepts of NEN evolution. Similarly, moving toward a morphomolecular classification is likely to be more relevant for patients with NETs. In parallel, gaining a better understanding of the cell of origin, evolutionary history, and the role of the microenvironment is essential for effectively tackling these diseases. The wide range of clinical behaviors displayed by NETs — from slow progression to metastatic — likely reflects an underappreciated heterogeneity in the NE cells of origin and their microenvironments. For example, pulmonary NETs can be found in different anatomic locations within the lung — peripheral or central (in the bronchi) — which are associated with distinct molecular features. It is very likely that these tumors arise from distinct NE cells depending on their location and exposure to different environmental stimuli. To accelerate the discovery of novel therapeutic targets, further comprehensive molecular studies are needed. Only such studies will guide researchers in testing biologically grounded therapeutic options that will improve patient outcomes and drive personalized therapy. The current trend toward agnostic oncology for rare cancers, which combines them based on single actionable molecular alterations or their common NE nature, is likely to fail without robust molecular basis.

## Figures and Tables

**Table 3:**
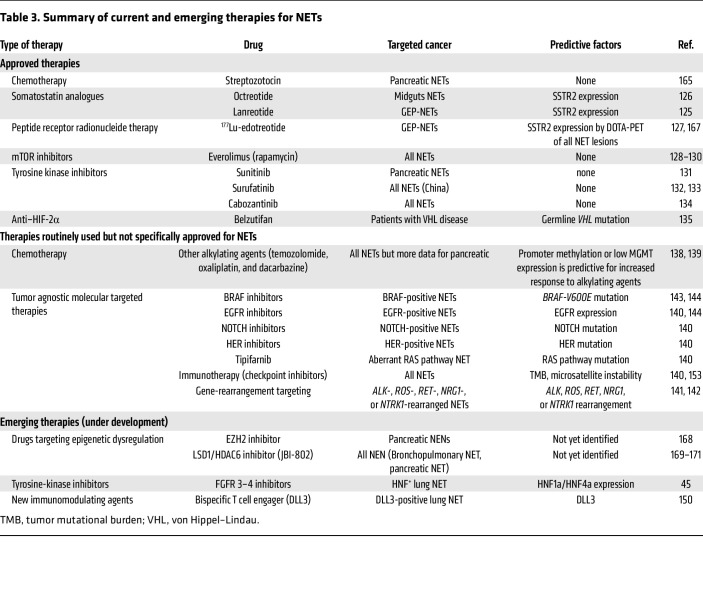
Summary of current and emerging therapies for NETs

**Table 2:**
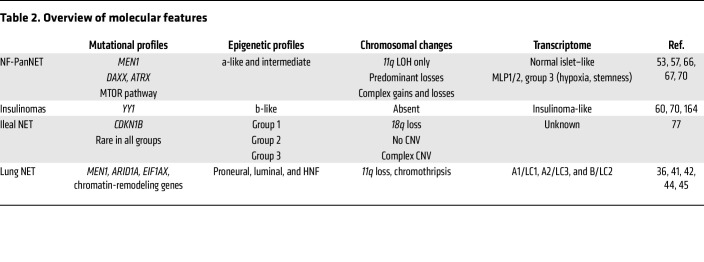
Overview of molecular features

**Table 1:**
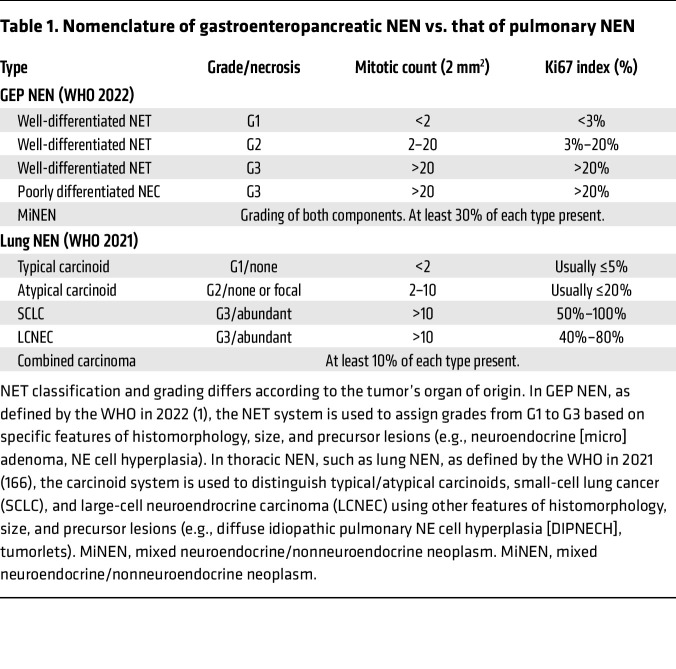
Nomenclature of gastroenteropancreatic NEN vs. that of pulmonary NEN

## Version 1 03/03/2025

Electronic publication

## References

[2] Perez-Ordoñez B (2018). Neuroendocrine carcinomas of the larynx and head and neck: challenges in classification and grading. Head Neck Pathol.

[3] Kim B (2019). Primary renal well-differentiated neuroendocrine tumors: report of six cases with an emphasis on the Ki-67 index and mitosis. Diagn Pathol.

[4] Kouba E, Cheng L (2016). Neuroendocrine tumors of the urinary bladder according to the 2016 World Health Organization Classification: molecular and clinical characteristics. Endocr Pathol.

[5] Goto K (2017). Low-grade neuroendocrine carcinoma of the skin (primary cutaneous carcinoid tumor) as a distinctive entity of cutaneous neuroendocrine tumors: a clinicopathologic study of 3 cases with literature review. Am J Dermatopathol.

[6] Amine MM (2017). Neuroendocrine testicular tumors: a systematic review and meta-analysis. Curr Urol.

[7] Dasari A (2017). Trends in the incidence, prevalence, and survival outcomes in patients with neuroendocrine tumors in the United States. JAMA Oncol.

[8] Alwan H (2020). Incidence trends of lung and gastroenteropancreatic neuroendocrine neoplasms in Switzerland. Cancer Med.

[9] Darbà J, Marsà A (2019). Exploring the current status of neuroendocrine tumours: a population-based analysis of epidemiology, management and use of resources. BMC Cancer.

[10] Masui T (2020). Recent epidemiology of patients with gastro-entero-pancreatic neuroendocrine neoplasms (GEP-NEN) in Japan: a population-based study. BMC cancer.

[11] Farooqui ZA, Chauhan A (2019). Neuroendocrine tumors in pediatrics. Glob Pediatr Health.

[12] Smith NJ (2021). Epidemiology and outcomes of primary pediatric lung malignancies: Updates from the SEER database. Am J Surg.

[13] Abou Saleh M (2019). Prevalence of small intestine carcinoid tumors: A US population-based study 2012-2017. Dig Dis Sci.

[14] Lal P (2020). Epidemiology of large bowel carcinoid tumors in the USA: a population-based national study. Dig Dis Sci.

[15] Giraldi L (2021). Risk factors for pancreas and lung neuroendocrine neoplasms: a case-control study. Endocrine.

[16] Kasajima A (2022). An analysis of 130 neuroendocrine tumors G3 regarding prevalence, origin, metastasis, and diagnostic features. Virchows Arch.

[17] Hermans BCM (2021). Unique metastatic patterns in neuroendocrine neoplasms of different primary origin. Neuroendocrinology.

[18] Fesinmeyer MD (2005). Differences in survival by histologic type of pancreatic cancer. Cancer Epidemiol Biomarkers Prev.

[19] Kim JY (2017). Alternative lengthening of telomeres in primary pancreatic neuroendocrine tumors is associated with aggressive clinical behavior and poor survival. Clin Cancer Res.

[20] Chou A (2018). ATRX loss is an independent predictor of poor survival in pancreatic neuroendocrine tumors. Hum Pathol.

[21] Riihimäki M (2016). The epidemiology of metastases in neuroendocrine tumors. Int J Cancer.

[22] Rekhtman N (2019). Stage IV lung carcinoids: spectrum and evolution of proliferation rate, focusing on variants with elevated proliferation indices. Mod Pathol.

[23] Pavel M (2020). Gastroenteropancreatic neuroendocrine neoplasms: ESMO Clinical Practice Guidelines for diagnosis, treatment and follow-up. Ann Oncol.

[24] https://www.enets.org/guidelines.html.

[25] Ricci C (2021). Sporadic non-functioning pancreatic neuroendocrine tumours: multicentre analysis. Br J Surg.

[26] Cattoni M (2018). Improvement in TNM staging of pulmonary neuroendocrine tumors requires histology and regrouping of tumor size. J Thorac Cardiovasc Surg.

[27] Merath K (2018). Nomogram predicting the risk of recurrence after curative-intent resection of primary non-metastatic gastrointestinal neuroendocrine tumors: An analysis of the U.S. Neuroendocrine Tumor Study Group. J Surg Oncol.

[28] Pulvirenti A (2019). Limited role of Chromogranin A as clinical biomarker for pancreatic neuroendocrine tumors. HPB (Oxford).

[29] Zaidi MY (2019). A novel validated recurrence risk score to guide a pragmatic surveillance strategy after resection of pancreatic neuroendocrine tumors: an international study of 1006 patients. Ann Surg.

[30] Sho S (2019). A prognostic scoring system for the prediction of metastatic recurrence following curative resection of pancreatic neuroendocrine tumors. J Gastrointest Surg.

[31] https://rarecancersgenomics.com/lungnenomics/.

[32] Mathian É (2024). Assessment of the current and emerging criteria for the histopathological classification of lung neuroendocrine tumours in the lungNENomics project. ESMO Open.

[33] Derks JL (2016). A population-based analysis of application of WHO nomenclature in pathology reports of pulmonary neuroendocrine tumors. J Thorac Oncol.

[34] Rekhtman N (2022). Lung neuroendocrine neoplasms: recent progress and persistent challenges. Mod Pathol.

[35] Merola E (2022). Radical resection in entero-pancreatic neuroendocrine tumors: recurrence-free survival rate and definition of a risk score for recurrence. Ann Surg Oncol.

[36] Fernandez-Cuesta L (2014). Frequent mutations in chromatin-remodelling genes in pulmonary carcinoids. Nat Commun.

[37] George J (2015). Comprehensive genomic profiles of small cell lung cancer. Nature.

[38] George J (2018). Integrative genomic profiling of large-cell neuroendocrine carcinomas reveals distinct subtypes of high-grade neuroendocrine lung tumors. Nat Commun.

[39] Machens A (2007). Age-related penetrance of endocrine tumours in multiple endocrine neoplasia type 1 (MEN1): a multicentre study of 258 gene carriers. Clin Endocrinol (Oxf).

[40] Kouvaraki MA (2002). Genotype-phenotype analysis in multiple endocrine neoplasia type 1. Arch Surg.

[41] Alcala N (2019). Integrative and comparative genomic analyses identify clinically relevant pulmonary carcinoid groups and unveil the supra-carcinoids. Nat Commun.

[42] Laddha SV (2019). Integrative genomic characterization identifies molecular subtypes of lung carcinoids. Cancer Res.

[43] Gabriel AAG (2020). A molecular map of lung neuroendocrine neoplasms. Gigascience.

[44] Domingo-Sabugo C (2024). Genomic analysis defines distinct pancreatic and neuronal subtypes of lung carcinoid. J Pathol.

[45] Davis E (2024). Enhancer landscape of lung neuroendocrine tumors reveals regulatory and developmental signatures with potential theranostic implications. Proc Natl Acad Sci U S A.

[46] Simbolo M (2019). Gene expression profiling of lung atypical carcinoids and large cell neuroendocrine carcinomas identifies three transcriptomic subtypes with specific genomic alterations. J Thorac Oncol.

[47] Centonze G (2023). Ascl1 and OTP tumour expressions are associated with disease-free survival in lung atypical carcinoids. Histopathology.

[48] Werr L TERT expression and clinical outcome in pulmonary carcinoids. J Clin Oncol.

[50] Papaxoinis G (2018). Clinical and pathologic characteristics of pulmonary carcinoid tumors in central and peripheral locations. Endocr Pathol.

[51] Jindal T (2011). Evaluation of the role of [18F]FDG-PET/CT and [68Ga]DOTATOC-PET/CT in differentiating typical and atypical pulmonary carcinoids. Cancer Imaging.

[52] Chan DLH (2017). Dual somatostatin receptor/FDG PET/CT imaging in metastatic neuroendocrine tumours: proposal for a novel grading scheme with prognostic significance. Theranostics.

[53] Scarpa A (2017). Whole-genome landscape of pancreatic neuroendocrine tumours. Nature.

[54] Jiao Y (2011). DAXX/ATRX, MEN1, and mTOR pathway genes are frequently altered in pancreatic neuroendocrine tumors. Science.

[55] Elsässer SJ (2011). Cancer. New epigenetic drivers of cancers. Science.

[56] Marinoni I (2014). Loss of DAXX and ATRX are associated with chromosome instability and reduced survival of patients with pancreatic neuroendocrine tumors. Gastroenterology.

[57] Lawrence B (2018). Recurrent loss of heterozygosity correlates with clinical outcome in pancreatic neuroendocrine cancer. NPJ Genom Med.

[58] Luchini C (2021). Alternative lengthening of telomeres (ALT) in pancreatic neuroendocrine tumors: ready for prime-time in clinical practice?. Curr Oncol Rep.

[59] Nguyen B (2022). Genomic characterization of metastatic patterns from prospective clinical sequencing of 25,000 patients. Cell.

[60] Cao Y (2013). Whole exome sequencing of insulinoma reveals recurrent T372R mutations in YY1. Nat Commun.

[61] Venizelos A (2021). The molecular characteristics of high-grade gastroenteropancreatic neuroendocrine neoplasms. Endocr Relat Cancer.

[62] Simon T (2022). DNA methylation reveals distinct cells of origin for pancreatic neuroendocrine carcinomas and pancreatic neuroendocrine tumors. Genome Med.

[63] Backman S (2024). The evolutionary history of metastatic pancreatic neuroendocrine tumours reveals a therapy driven route to high-grade transformation. J Pathol.

[64] Botling J (2020). High-grade progression confers poor survival in pancreatic neuroendocrine tumors. Neuroendocrinology.

[65] Raj N (2018). Real-time genomic characterization of metastatic pancreatic neuroendocrine tumors has prognostic implications and identifies potential germline actionability. JCO Precis Oncol.

[66] Sadanandam A (2015). A cross-species analysis in pancreatic neuroendocrine tumors reveals molecular subtypes with distinctive clinical, metastatic, developmental, and metabolic characteristics. Cancer Discov.

[67] Young K (2021). Immune landscape, evolution, hypoxia-mediated viral mimicry pathways and therapeutic potential in molecular subtypes of pancreatic neuroendocrine tumours. Gut.

[68] Di Domenico A (2017). Genetic and epigenetic drivers of neuroendocrine tumours (NET). Endocr Relat Cancer.

[69] Cejas P (2019). Enhancer signatures stratify and predict outcomes of non-functional pancreatic neuroendocrine tumors. Nat Med.

[70] Di Domenico A (2020). Epigenetic landscape of pancreatic neuroendocrine tumours reveals distinct cells of origin and means of tumour progression. Commun Biol.

[71] Swarts DRA (2014). MEN1 gene mutation and reduced expression are associated with poor prognosis in pulmonary carcinoids. J Clin Endocrinol Metab.

[72] van den Broek MFM (2021). Well-differentiated bronchopulmonary neuroendocrine tumors: more than one entity. J Thorac Oncol.

[73] Lecomte P (2018). Histologically proven bronchial neuroendocrine tumors in MEN1: A GTE 51-case cohort study. World J Surg.

[74] Thevenon J (2015). Unraveling the intrafamilial correlations and heritability of tumor types in MEN1: a Groupe d’étude des Tumeurs Endocrines study. Eur J Endocrinol.

[75] Francis JM (2013). Somatic mutation of CDKN1B in small intestine neuroendocrine tumors. Nat Genet.

[76] Zhang Z (2020). Patterns of chromosome 18 loss of heterozygosity in multifocal ileal neuroendocrine tumors. Genes Chromosomes Cancer.

[77] Karpathakis A (2016). Prognostic impact of novel molecular subtypes of small intestinal neuroendocrine tumor. Clin Cancer Res.

[78] Candeli N, Dayton T (2024). Investigating pulmonary neuroendocrine cells in human respiratory diseases with airway models. Dis Model Mech.

[79] Quach H (2024). Early human fetal lung atlas reveals the temporal dynamics of epithelial cell plasticity. Nat Commun.

[80] Kuo CS (2022). Neuroendocrinology of the lung revealed by single-cell RNA sequencing. Elife.

[81] He P (2022). A human fetal lung cell atlas uncovers proximal-distal gradients of differentiation and key regulators of epithelial fates. Cell.

[82] Guha A (2012). Neuroepithelial body microenvironment is a niche for a distinct subset of Clara-like precursors in the developing airways. Proc Natl Acad Sci U S A.

[83] Kuhnen C, Winter BU (2006). [EGFR-expression in pulmonary neuroendocrine cell hyperplasia]. Pathologe.

[84] Kawasaki K (2023). Neuroendocrine neoplasms of the lung and gastrointestinal system: convergent biology and a path to better therapies. Nat Rev Clin Oncol.

[85] Klöppel G (2019). [Neuroendocrine neoplasms: Two families with distinct features unified in one classification (German version)]. Pathologe.

[86] Lisa Sikkema (2023). An integrated cell atlas of the lung in health and disease. Nat Med.

[87] Sutherland KD (2011). Cell of origin of small cell lung cancer: inactivation of Trp53 and Rb1 in distinct cell types of adult mouse lung. Cancer Cell.

[88] Lázaro S (2019). Differential development of large-cell neuroendocrine or small-cell lung carcinoma upon inactivation of 4 tumor suppressor genes. Proc Natl Acad Sci U S A.

[89] Kawasaki K (2020). An organoid biobank of neuroendocrine neoplasms enables genotype-phenotype mapping. Cell.

[90] Griger J (2023). An integrated cellular and molecular model of gastric neuroendocrine cancer evolution highlights therapeutic targets. Cancer Cell.

[91] Cives M (2019). The tumor microenvironment in neuroendocrine tumors: biology and therapeutic implications. Neuroendocrinology.

[92] Marion-Audibert A-M (2003). Low microvessel density is an unfavorable histoprognostic factor in pancreatic endocrine tumors. Gastroenterology.

[93] Terris B (1998). Expression of vascular endothelial growth factor in digestive neuroendocrine tumours. Histopathology.

[94] Bischoff P (2022). The single-cell transcriptional landscape of lung carcinoid tumors. Int J Cancer.

[95] Ferrata M (2019). PD-L1 expression and immune cell infiltration in gastroenteropancreatic (GEP) and non-gep neuroendocrine neoplasms with high proliferative activity. Front Oncol.

[96] Roberts JA (2017). Expression of PD-1 and PD-L1 in poorly differentiated neuroendocrine carcinomas of the digestive system: a potential target for anti-PD-1/PD-L1 therapy. Hum Pathol.

[97] Chen Z (2024). The prognostic and therapeutic value of the tumor microenvironment and immune checkpoints in pancreatic neuroendocrine neoplasms. Sci Rep.

[98] Cai L (2019). Role of tumor-associated macrophages in the clinical course of pancreatic neuroendocrine tumors (PanNETs). Clin Cancer Res.

[99] Werner W (2023). Intratumoral dendritic cells and T cells predict survival in gastroenteropancreatic neuroendocrine neoplasms. Endocr Relat Cancer.

[100] Combes AJ (2023). Defining and using immune archetypes to classify and treat cancer. Nat Rev Cancer.

[101] Combes AJ (2022). Discovering dominant tumor immune archetypes in a pan-cancer census. Cell.

[102] Furukawa T (2021). Ki-67 labeling index variability between surgically resected primary and metastatic hepatic lesions of gastroenteropancreatic neuroendocrine neoplasms. Int J Surg Pathol.

[103] Xu M (2022). Evolutionary trajectories of primary and metastatic pancreatic neuroendocrine tumors based on genomic variations. Genes (Basel).

[104] Zhao J (2001). Genomic imbalances in the progression of endocrine pancreatic tumors. Genes Chromosomes Cancer.

[105] Alvarez MJ (2018). A precision oncology approach to the pharmacological targeting of mechanistic dependencies in neuroendocrine tumors. Nat Genet.

[106] Greenberg J (2022). Metastatic pancreatic neuroendocrine tumors feature elevated T cell infiltration. JCI Insight.

[107] Scott AT (2020). Gene expression signatures identify novel therapeutics for metastatic pancreatic neuroendocrine tumors. Clin Cancer Res.

[108] Karpathakis A (2017). Progressive epigenetic dysregulation in neuroendocrine tumour liver metastases. Endocr Relat Cancer.

[109] Ferone G (2020). Cells of origin of lung cancers: lessons from mouse studies. Genes Dev.

[110] Ouadah Y (2019). Rare pulmonary neuroendocrine cells are stem cells regulated by Rb, p53, and notch. Cell.

[111] Park JW (2018). Reprogramming normal human epithelial tissues to a common, lethal neuroendocrine cancer lineage. Science.

[112] Balanis NG (2019). Pan-cancer convergence to a small-cell neuroendocrine phenotype that shares susceptibilities with hematological malignancies. Cancer Cell.

[113] Quintanal-Villalonga Á (2020). Lineage plasticity in cancer: a shared pathway of therapeutic resistance. Nat Rev Clin Oncol.

[114] Davies AH (2018). Cellular plasticity and the neuroendocrine phenotype in prostate cancer. Nat Rev Urol.

[115] Marcoux N (2019). EGFR-mutant adenocarcinomas that transform to small-cell lung cancer and other neuroendocrine carcinomas: clinical outcomes. J Clin Oncol.

[116] Beltran H (2014). Aggressive variants of castration-resistant prostate cancer. Clin Cancer Res.

[117] Beltran H (2016). Divergent clonal evolution of castration-resistant neuroendocrine prostate cancer. Nat Med.

[118] Mu P (2017). SOX2 promotes lineage plasticity and antiandrogen resistance in TP53- and RB1-deficient prostate cancer. Science.

[119] Cancer Genome Atlas Research Network (2014). Comprehensive molecular profiling of lung adenocarcinoma. Nature.

[120] Swanton C (2024). Embracing cancer complexity: Hallmarks of systemic disease. Cell.

[121] Rekhtman N Chromothripsis-mediated small cell lung carcinoma. Cancer Discov.

[122] Cros J (2021). Specific genomic alterations in high-grade pulmonary neuroendocrine tumours with carcinoid morphology. Neuroendocrinology.

[123] Hackeng WM (2020). Alternative lengthening of telomeres and differential expression of endocrine transcription factors distinguish metastatic and non-metastatic insulinomas. Endocr Pathol.

[124] Saghafinia S (2021). Cancer cells retrace a stepwise differentiation program during malignant progression. Cancer Discov.

[125] Caplin ME (2014). Lanreotide in metastatic enteropancreatic neuroendocrine tumors. N Engl J Med.

[126] Arnold R (2009). Placebo-controlled, double-blind, prospective, randomized study on the effect of octreotide LAR in the control of tumor growth in patients with metastatic neuroendocrine midgut tumors: a report from the PROMID Study Group. J Clin Oncol.

[127] Strosberg J (2017). Phase 3 Trial of ^177^Lu-dotatate for midgut neuroendocrine tumors. N Engl J Med.

[128] Pavel ME (2012). Everolimus plus octreotide long-acting repeatable for the treatment of advanced neuroendocrine tumours associated with carcinoid syndrome (RADIANT-2): a randomised, placebo-controlled, phase 3 study. Lancet.

[129] Yao JC (2016). Everolimus for the treatment of advanced pancreatic neuroendocrine tumors: overall survival and circulating biomarkers from the randomized, phase III RADIANT-3 study. J Clin Oncol.

[130] Yao JC (2016). Everolimus for the treatment of advanced, non-functional neuroendocrine tumours of the lung or gastrointestinal tract (RADIANT-4) a randomised, placebo-controlled, phase 3 study. Lancet.

[131] Raymond E (2011). Sunitinib malate for the treatment of pancreatic neuroendocrine tumors. N Engl J Med.

[132] Xu J (2020). Surufatinib in advanced pancreatic neuroendocrine tumours (SANET-p): a randomised, double-blind, placebo-controlled, phase 3 study. Lancet Oncol.

[133] Xu J (2020). Surufatinib in advanced extrapancreatic neuroendocrine tumours (SANET-ep): a randomised, double-blind, placebo-controlled, phase 3 study. Lancet Oncol.

[134] Chan JA Phase 3 trial of cabozantinib to treat advanced neuroendocrine tumors. N Engl J Med.

[135] Else T (2024). Belzutifan for von Hippel-Lindau disease: pancreatic lesion population of the phase 2 LITESPARK-004 study. Clin Cancer Res.

[136] Ballman KV (2015). Biomarker: predictive or prognostic?. J Clin Oncol.

[137] Hu C, Dignam JJ (2019). Biomarker-driven oncology clinical trials: Key design elements, types, features, and practical considerations. JCO Precis Oncol.

[138] Kunz PL (2023). Randomized study of temozolomide or temozolomide and capecitabine in patients with advanced pancreatic neuroendocrine tumors (ECOG-ACRIN E2211). J Clin Oncol.

[139] Walter T (2024). Oxaliplatin-based versus alkylating agent in neuroendocrine tumors according to the O6-Methylguanine-DNA Methyltransferase Status: A Randomized Phase II Study (MGMT-NET). J Clin Oncol.

[140] Boilève A (2023). Molecular profiling and target actionability for precision medicine in neuroendocrine neoplasms: real-world data. Eur J Cancer.

[141] Liu N (2020). A case of primary pulmonary atypical carcinoid with EML4-ALK rearrangement. Cancer Biol Ther.

[142] Wang VE (2017). A case of metastatic atypical neuroendocrine tumor with *ALK* translocation and diffuse brain metastases. Oncologist.

[143] Elvebakken H (2023). Impact of KRAS and BRAF mutations on treatment efficacy and survival in high-grade gastroenteropancreatic neuroendocrine neoplasms. J Neuroendocrinol.

[144] Dayton TL (2023). Druggable growth dependencies and tumor evolution analysis in patient-derived organoids of neuroendocrine neoplasms from multiple body sites. Cancer Cell.

[145] Hu W (2024). A rare case report of a primary lung cancer comprising adenocarcinoma and atypical carcinoid tumor, with the carcinoid component harboring *EML4-ALK* rearrangement. Transl Lung Cancer Res.

[146] Armstrong S (2024). Lung neuroendocrine neoplasms harboring ALK, RET and other receptor tyrosine kinase fusions: Spectrum of pathologic features from conventional neuroendocrine carcinomas to unusual carcinoid-type tumors with focal mucin or adenocarcinoma components. Laboratory Investigation.

[147] Gerard L (2021). ctDNA in neuroendocrine carcinoma of gastroenteropancreatic origin or of unknown primary: The CIRCAN-NEC pilot study. Neuroendocrinology.

[148] Boons G (2022). Longitudinal copy-number alteration analysis in plasma cell-free DNA of neuroendocrine neoplasms is a novel specific biomarker for diagnosis, prognosis, and follow-up. Clin Cancer Res.

[149] Xie H (2019). Expression of delta-like protein 3 is reproducibly present in a subset of small cell lung carcinomas and pulmonary carcinoid tumors. Lung Cancer.

[150] Cooper AJ (2024). First report of response to tarlatamab in a patient with DLL3-positive pulmonary carcinoid: case report. JTO Clin Res Rep.

[151] Baudin E (2024). Treatment of advanced BP-NETS with lanreotide autogel/depot vs placebo: the phase III SPINET study. Endocr Relat Cancer.

[152] Cella CA (2023). Cabozantinib in neuroendocrine tumors: tackling drug activity and resistance mechanisms. Endocr Relat Cancer.

[153] Capdevila J (2023). Durvalumab plus tremelimumab for the treatment of advanced neuroendocrine neoplasms of gastroenteropancreatic and lung origin. Nat Commun.

[154] Owen DH (2023). A phase II clinical trial of nivolumab and temozolomide for neuroendocrine neoplasms. Clin Cancer Res.

[155] Klein O (2020). Immunotherapy of ipilimumab and nivolumab in patients with advanced neuroendocrine tumors: a subgroup analysis of the CA209-538 clinical trial for rare cancers. Clin Cancer Res.

[156] Yao JC (2021). Spartalizumab in metastatic, well/poorly-differentiated neuroendocrine neoplasms. Endocr Relat Cancer.

[157] Puccini A (2020). Comprehensive genomic profiling of gastroenteropancreatic neuroendocrine neoplasms (GEP-NENs). Clin Cancer Res.

[158] Touat M (2020). Mechanisms and therapeutic implications of hypermutation in gliomas. Nature.

[159] Morano F (2022). Temozolomide followed by combination with low-dose ipilimumab and nivolumab in patients with microsatellite-stable, O^6^-methylguanine-DNA methyltransferase-silenced metastatic colorectal cancer: The MAYA Trial. J Clin Oncol.

[160] Cousin S (2022). REGOMUNE: Phase II study of regorafenib plus avelumab in solid tumors—Results of the gastroenteropancreatic neuroendocrine carcinomas (GEP-NEC) cohort. J Clin Oncol.

[161] Al-Toubah T (2024). Phase II study of pembrolizumab and lenvatinib in advanced well-differentiated neuroendocrine tumors. ESMO Open.

[162] Rindi G (2018). A common classification framework for neuroendocrine neoplasms: an International Agency for Research on Cancer (IARC) and World Health Organization (WHO) expert consensus proposal. Mod Pathol.

[163] Galon J (2006). Type, density, and location of immune cells within human colorectal tumors predict clinical outcome. Science.

[164] Hong X (2020). Whole-genome sequencing reveals distinct genetic bases for insulinomas and non-functional pancreatic neuroendocrine tumours: leading to a new classification system. Gut.

[165] Capdevila J (2022). Streptozotocin, 1982-2022: forty years from the FDA’s approval to treat pancreatic neuroendocrine tumors. Neuroendocrinology.

[167] Singh S (2024). [^177^Lu]Lu-DOTA-TATE plus long-acting octreotide versus high‑dose long-acting octreotide for the treatment of newly diagnosed, advanced grade 2-3, well-differentiated, gastroenteropancreatic neuroendocrine tumours (NETTER-2): an open-label, randomised, phase 3 study. Lancet.

[168] April-Monn SL (2021). EZH2 inhibition as new epigenetic treatment option for pancreatic neuroendocrine neoplasms (PanNENs). Cancers (Basel).

[169] Jamison JK (2024). Entinostat in patients with relapsed or refractory abdominal neuroendocrine tumors. Oncologist.

[170] Balasubramaniam S (2018). Phase I trial of belinostat with cisplatin and etoposide in advanced solid tumors, with a focus on neuroendocrine and small cell cancers of the lung. Anticancer Drugs.

[171] Hollebecque A (2021). Phase I study of lysine-specific demethylase 1 inhibitor, CC-90011, in patients with advanced solid tumors and relapsed/refractory non-Hodgkin lymphoma. Clin Cancer Res.

